# Severe Hair Loss of the Scalp due to a Hair Dye Containing Para phenylenediamine

**DOI:** 10.5402/2011/947284

**Published:** 2011-06-07

**Authors:** Waka Ishida, Teruhiko Makino, Tadamichi Shimizu

**Affiliations:** ^1^Department of Dermatology, Niigata Central Hospital, Jyoetsu, Niigata 943-0192, Japan; ^2^Department of Dermatology, Graduate School of Medicine and Pharmaceutical Sciences, University of Toyama, Sugitani, Toyama 930-0194, Japan

## Abstract

We report the case of a 41-year-old female showing severe hair loss approximately 90% after the use of a hair dye. These symptoms developed six days after the use of a hair dye containing PPD. A patch test showed a (++) reaction at 48 h to 1% PPD in petrolatum, whereas all metals and white petrolatum were negative. She was therefore diagnosed with contact dermatitis due to PPD, resulting in hair loss. The skin lesions gradually improved after starting treatment with the systemic corticosteroids. The possibility that allergic contact dermatitis from hair dyes may be responsible for telogen effluvium should always be considered in a patient with increased hair loss.

## 1. Case Report

**Figure 1:**
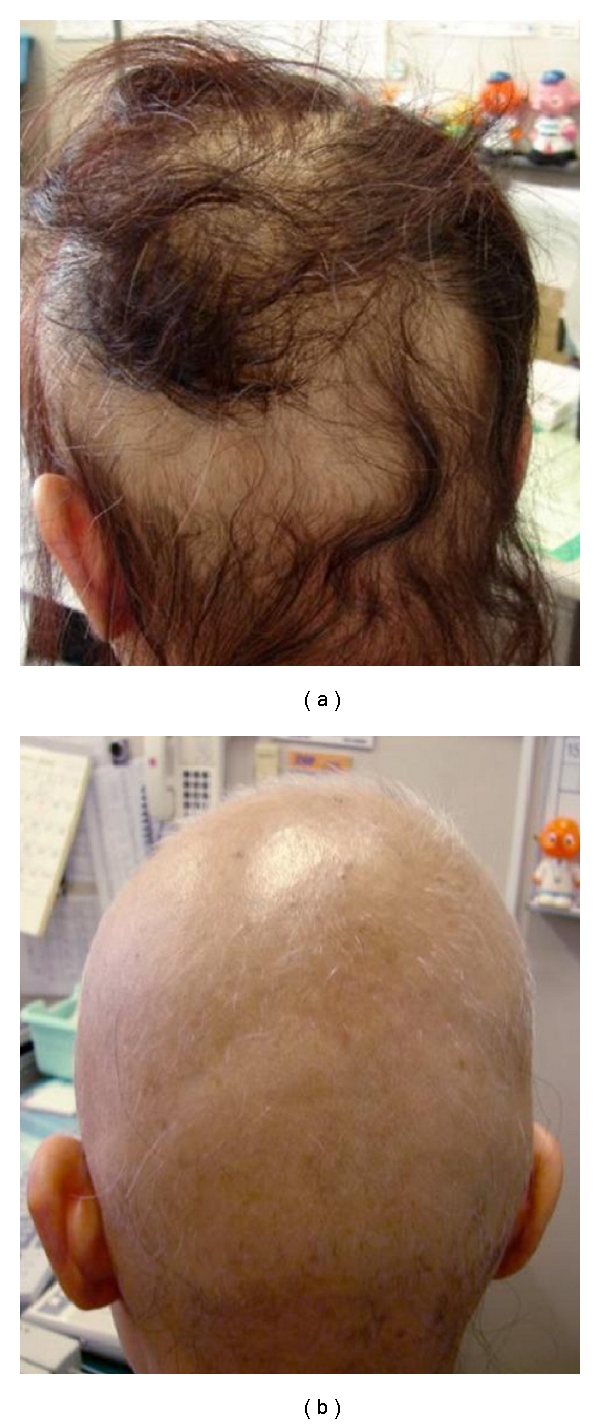
(a) A female with an allergic reaction to hair dye with severe hair loss on the scalp. This picture was taken 6 days after she had colored her hair. (b) The hair loss spread to approximately 90% of the scalp 2 months later. Partial white hair regrowth was also observed.

A 41-year-old female was referred because of edema on the face and hair loss with severe itching of the scalp. The itching of the scalp started 1 day after the use of a hair dye containing PPD, and hair loss symptoms developed 6 days after the use of the hair dye (Figure 1(a)). Hair loss had spread to approximately 90% of the scalp 4 months later (Figure 1(b)). Detailed anamneses revealed that the patient had started to use hair dye 5 years prior to the current presentation and had developed sprier itching of the scalp 1 to 2 days after her hair was dyed at a hair dresser's several months ago. All parameters examined during a blood test were within the normal limits. There was no history of associated dermatitis. A patch test showed a (++) reaction at 48 h and 72 h to 1% PPD in petrolatum. She was therefore diagnosed with contact dermatitis due to PPD, resulting in hair loss. The skin lesions gradually improved after starting treatment with the systemic corticosteroids. The patient has not had a recurrence for 18 months since she stopped using the hair dye in question.

## 2. Discussion

Several cases of severe facial and scalp dermatitis have been reported following the use of permanent hair dyes, and these dyes often contain paraphenylendiamine (PPD). PPD is known to be the most frequent contact allergen found in hair dyes [1]. The possibility that allergic contact dermatitis from hair dyes may be responsible for telogen effluvium should always be considered in a patient with increased hair loss [2]. This report presents the case of a patient who experienced severe hair loss after the use of a hair dye containing PPD. We concluded that the explanation for the severe hair loss in our case was a concurrent sensitization and allergic reaction to PPD, as the product had been applied repeatedly over a long period of time.

Hair dye reactions are usually diagnosed by the patients themselves, and epidermological studies found a range between 0.1% and 1% of the patients sensitized to PPD [3, 4]. The most frequent symptoms tend to be erythema of the face, scalp, and ears following the use of permanent hair dyes containing PPD. Severe facial and scalp dermatitis with PPD has been reported in several cases, with symptoms of edema, suppuration, and ulceration of the face, scalp and eyelids [5]. The reports of hair loss in the scalp due to hair dyes containing PPD is rare, and there have been only 2 reported cases of hair loss in the scalp due to a PPD allergy associated with dermatitis in a consumer-based study [5]. Wachsmuth and Wilkison reported a case of eyelash loss due to an allergic reaction to PPD in mascara [6].

Sosted et al. reported that many cases of PPD allergy go unreported, probably because the patient is able to make the connection between the reaction and the hair dye and therefore does not seek medical advice. Our case had a severe face edematous reaction, with approximately 90% hair loss that required treatment with systemic corticosteroids.

## 3. Conclusions

Many cases of dermatitis due to hair dye may be overlooked, but severe dermatitis may sometimes last for more than 3 weeks [5]. Therefore, dermatologists need to pay more attention to hair dye allergies.

##  Conflict of Interests

The authors state no conflict of interests.
